# Uniting Statistical and Individual-Based Approaches for Animal Movement Modelling

**DOI:** 10.1371/journal.pone.0099938

**Published:** 2014-06-30

**Authors:** Guillaume Latombe, Lael Parrott, Mathieu Basille, Daniel Fortin

**Affiliations:** 1 School of Biological Sciences, Monash University, Clayton, Victoria, Australia; 2 Département de Géographie, Université de Montréal, Montréal, Québec, Canada; 3 Earth and Environmental Sciences and Biology Units, The University of British Columbia, Kelowna, British Columbia, Canada; 4 Chaire de recherche industrielle CRSNG-Université Laval en sylviculture et faune, Département de biologie, Université Laval, Québec, Québec, Canada; 5 Fort Lauderdale Research and Education Center, University of Florida, Fort Lauderdale, Florida, United States of America; Cinvestav-Merida, Mexico

## Abstract

The dynamic nature of their internal states and the environment directly shape animals' spatial behaviours and give rise to emergent properties at broader scales in natural systems. However, integrating these dynamic features into habitat selection studies remains challenging, due to practically impossible field work to access internal states and the inability of current statistical models to produce dynamic outputs. To address these issues, we developed a robust method, which combines statistical and individual-based modelling. Using a statistical technique for forward modelling of the IBM has the advantage of being faster for parameterization than a pure inverse modelling technique and allows for robust selection of parameters. Using GPS locations from caribou monitored in Québec, caribou movements were modelled based on generative mechanisms accounting for dynamic variables at a low level of emergence. These variables were accessed by replicating real individuals' movements in parallel sub-models, and movement parameters were then empirically parameterized using Step Selection Functions. The final IBM model was validated using both k-fold cross-validation and emergent patterns validation and was tested for two different scenarios, with varying hardwood encroachment. Our results highlighted a functional response in habitat selection, which suggests that our method was able to capture the complexity of the natural system, and adequately provided projections on future possible states of the system in response to different management plans. This is especially relevant for testing the long-term impact of scenarios corresponding to environmental configurations that have yet to be observed in real systems.

## Introduction

Predictive models of animal distribution are central to many fields of theoretical and applied ecology, and wildlife conservation and management strongly rely on such models, *e.g.*, [1]–[4]. Most models of animal distribution, or of the underlying mechanisms such as habitat selection and movements, are based on the response of individuals to habitat features. These features can be resources and modifying covariates [5], such as food or water availability, land cover types, slope, elevation, snow, and predation risk [6]–[8], which can further interact between each other (such as food quality and abundance [9]). However, the response of animals to particular habitat attributes may be affected by other dynamic factors over time. For example plant selection by herbivores can be altered by variations in group size and predation risk [10]. Working memory is such that the accuracy of the perception of visited high quality patches may vary over time [11], [12]. Lastly, the internal state of the animals, such as stored energy and gut fill, can also influence herbivores' grazing strategies [13]. Internal states have also been shown to be an essential component of animal's movements, along with motion capacity and navigation capacity [14].

The dynamic nature of internal states and habitat features, and its importance to explain animal's behaviour, is therefore broadly recognized [15]. Moreover, statistical techniques have the ability to take internal state variables into account. For example, internal state variables can be used as interaction terms with other external variables in Step Selection Functions (SSF) [16]. However, internal states have been accounted for in only a small proportion of habitat selection studies, *e.g.*, [17]–[20]. Indeed, they remain hardly usable to create predictive models using statistical techniques, for two reasons. First, whereas remote sensing and GPS technology provide a detailed assessment of habitat features and animal movement, keeping track of the internal state of the animal or its environment remains a challenge, because it would require costly or unpractical field work. Second, statistical models of habitat selection used for predicting the spatial distribution of animals generally produce static outputs which cannot represent the emergent properties of the natural system created by the dynamic nature of these variables.

Individual-based models (IBM – also called agent-based models, ABM) represent an alternative to statistical modelling as predictive models, and can easily integrate individuals' internal states in their implementation, *e.g.*, [21]. IBMs are modelling tools that represent distinct entities, such as individual animals, at relatively small spatial and temporal scales with respect to the studied system, and allow for the emergence of phenomena at broader scales and higher hierarchical levels (*e.g.* at the population level [22]). In the semantic of IBMs, internal states are related to generative mechanisms, which are defined as “the internal organization and processes that generate the system's responses” [4]. By using generative mechanisms as traits (or rules) governing the individuals' behaviours in the model, the resulting IBM can be valid for a large domain of applicability and account for plasticity in the individuals' behaviours. Modelling the generative mechanisms governing an individual's behaviour at the lowest level of emergence produces a model that can be used to predict how individuals might respond to changes in their environment, as well as any emergent patterns at higher levels of organization [23], and such IBMs are therefore useful tools for predicting the impacts of management plans on the dynamics of natural systems.

A common practice to parameterize generative mechanisms involves the iterative adjustment of IBM parameters so that the output of the model reproduces some patterns observed in the real system, *i.e.* an inverse modelling technique. However, although some frameworks, such as Pattern-oriented modelling (POM) [22], [24], [25], have been developed to perform inverse modelling while avoiding over-complex models, they require that the number of patterns is not disproportionately low with respect to the number of parameters to inversely estimate [23]. They also require extensive simulation runs to refine the parameters, which may be impractical for long simulations. On the other hand, forward modelling consists in assessing the trait's parameters directly from the available data without relying on the IBM's outputs, using, for example, statistical techniques. Forward modelling therefore allows to assess any number of parameters with an accuracy independent from the number of patterns. The IBM's outputs can then be used for validation, without also serving for model calibration, thus increasing the robustness of the model. While forward modelling is preferable to inverse modelling when practical, it faces the same limitations as statistical modelling with respect to keeping track of internal variables, which are usually overcome using inverse modelling.

In this study, we show how a combination of rigorous statistical modelling with individual-based methods allows to overcome their respective limitations and we apply these methods to predict how changing patterns of heterogeneity on a landscape affect the spatial repartition of animals across multiple spatiotemporal scales. To this end, we designed an IBM of woodland caribou (*Rangifer tarandus caribou*, L.) movements in managed boreal forest in Québec. Woodland caribou populations are declining in most of Canada, and this species is now considered as threatened in the Canadian boreal forest [26]. Anthropogenic activities are most likely the main cause of this situation, as evidence shows a strong correlation between the northward advancement of the forest harvesting front, and the southern limit of woodland caribou occupancy [27].

We used the framework of levels of emergence [23] to identify generative mechanisms allowing for the model to be valid for different configurations of the landscape. We related the generative mechanisms to energetic requirements, spatial memory, and habitat characteristics, to represent the trade-offs between costs and benefits of moving, and modelled accordingly the movement of caribou monitored by GPS telemetry based on a Step Selection Function calibrated by forward modelling. To keep track of internal state variables for parameterizing the SSF, we computationally generated these data at the individual level by replicating the real individuals' recorded behaviour in a simulation and simultaneously applying independently parameterized submodels related to the desired variables. Using statistical techniques such as SSFs as an IBM's trait also has the advantage of offering validation procedures usually not used in the validation of IBMs, such as k-fold cross-validation , *e.g.*, [28], [29], thus increasing the model's robustness. We also validated the model by comparing patterns at higher levels of emergence (habitat selection and home range size) produced by the model and observed in the real system. Finally, we ran simulations for the current landscape of the study area and for a hypothetical landscape with hardwood encroachment, and observed a functional response in habitat selection by the simulated individuals, demonstrating that IBMs based on generative mechanisms allow for the prediction of animals' plastic reactions to changes in the environment.

## Development of the movement model

### Ethics Statement

The study was carried out in strict accordance with the recommendations in the Guide to the Care and Use of Experimental Animals of the Canadian Council on Animal Care. The protocol was approved by an Institutional Animal Care and Use Committee – Comité de protection des animaux of the Université Laval Permit Number: 2008026-3. Caribou collaring was done with individuals under physical restraint. All efforts were made to minimize stress and suffering. Collaring was performed by members of the Quebec Ministry of Natural Resources, and no permit was required for the captures. Woodland caribou is considered as threatened in the Canadian boreal forest by the Committee on the Status of Endangered Wildlife in Canada [26].

### Study area and GPS monitoring

The study area is located in the Côte-Nord region (50*°*N to 52*°*N, 68*°*W to 71*°*W) of Québec, Canada. An aerial survey conducted over the study area in March 2007 revealed that caribou density was 1.9 individuals/100 km^2^ [30]. We concentrated our case study on the winter period, from December 28th to April 15th [31], because it represents a time of high stress for this species. Heavy snow increases predation risk, as well as the energy allocated to locomotion and foraging [32]. Twenty-seven caribou were monitored using GPS collars (Lotek Engineering, Newmarket, ON) or ARGOS/GPS collars (Telonics, Mesa, AZ), from 2005 to 2009, during which each caribou was followed for an average of 19 months. Radio-collars were scheduled to record a location every 4 hours.

We used Landsat Thematic Mapper images taken in 2000 with a 25-m resolution grid describing landcover classes found in the study area (Natural Resources Canada, Canadian Forest Service, Laurentian Forestry Centre). Satellite images were composed of 48 land cover classes, which were reclassified into 12 classes for which we had lichen biomass estimates [33]: fixed open area, burned area, water body, heath without lichen, heath with lichen, wetlands, regenerating mixed forest, regenerating coniferous stand, open conifer stand without lichen, dense mature conifer forest, open conifer stand with lichen, mixed/deciduous forest. We added 3 classes, namely regenerating cut (5–20 years after a forest cut), recent cut (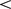
5 years after a forest cut), and road, based on data provided by the forestry companies operating in the region. These three classes and the burned area class were updated on a yearly basis. For example, after 5 years, a recent cut was reclassified as a regenerating cut, and recent cuts were added every year. As a result, the spatial distribution of these three classes was not constant over the years. A digital elevation model at the scale of 1∶20 000 was then used to estimate elevation and slope in the study area.

### Generative mechanisms and variables

#### Identifying the generative mechanism to allow for generalization

Movements were modelled by a biased correlated random walk (BCRW). Three broad kinds of biases were considered: the geometric characteristics of the observed movements, *i.e.* the step length (SL) and the turning angle (TA, *i.e.* the angle from the previous step to the current step) distributions, the landcover types, and the costs and benefits of moving. These three biases can be ordered according to the level of emergence framework [23]. Indeed, if individuals select or avoid some landcover types, different amounts and spatial configurations of these landcover types should give rise to different SL and TA distributions. The approach based on landcover types thus has a lower level of emergence than the approach based on SL and TA. Similarly, if individuals move according to some trade-off between costs and benefits related to their energetics and predation risk (for example, if percentage of canopy cover represents a shelter to hide from predators, a well fed individual may favour this kind of environment even if it provides few resources; conversely, a hungry individual may select risky environments if it provides resources [34]), we hypothesized that this trade-off could translate into different selections of the various landcover types according to their amounts and spatial configurations, a phenomenon known as functional response [35]. A modelling approach based on the costs and benefits of moving thus has a lower level of emergence than the approach based on the selection of specific landcover types. It can therefore be considered as a generative mechanism that should allow the model to be valid for different environmental conditions.

Following Latombe et al. [23], we define the *intended domain of applicability* as the whole set of conditions for which the model is supposed to be used, with respect to the *effective domain of applicability*, which is the set of conditions for which the model is valid in practice. If the level of emergence of the IBM's process is too high, the process will be specific to the particular environmental conditions corresponding to the data used for parameterization, and the effective domain of applicability will be smaller than the intended domain of applicability, which is a case of overfitting. On the other hand, using a generative mechanism at a lower level of emergence, such as the trade-off between costs and benefits of moving as used in this study, will produce a model able to generalize to a larger set of environmental conditions than the ones used for parameterization, thus preventing overfitting.

#### Variables corresponding to the generative mechanism

To represent the trade-offs between the costs and benefits of moving, we included variables corresponding or related to habitat features and internal state variables, estimated for each step in the movement model. Vegetation biomass was observed to influence habitat selection by caribou [36]. As proxies for vegetation biomass, we used the percentage of canopy cover at the end of the step *Cover* and the 2D gradient of percentage of canopy cover at the end of the step *Edge*, computed over a 3
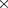
3 Moore neighbourhood (a high value of *Edge* of a cell represents a transition from one density of canopy cover to another [37]). The distance to human-induced habitat edges was shown to influence caribou movements and the resulting spatial distribution [38]. We thus considered in our analyses the angle and distance to the closest road (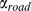 and *D_road_*), recent cut (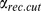 and 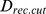), and regenerating cut (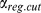 and 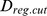).

Caribou behaviour is influenced by forage distribution [39]. The short-term functional response of lichen consumption *X* was modelled using a Michaelis-Menten equation (1) [40]:
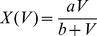
(1)where *a* is the maximum rate of consumption, *b* is the foraging efficiency, *i.e.* the resource biomass for which intake is one-half of the maximum rate, and *V* is the resource biomass of the cell. *a* was set to 61.3 g/day per kg body weight [41]. *b* was set to 40 g/m^2^ [40]. *V* varied according to the land cover type, and was taken from [33]. *X* was then converted into energy gains *G*, with a rate of 7.79 kJ/g (1.86 kcal/g) [41], in order to be more easily compared with energy expenses due to displacements.

For each 4-hour step, energy expenditures *L* are computed by summing the basal metabolic rate for a time step, the energy expenses due to the travelled distance, and the energy expenses due to the difference of altitude along a step [42], [43].

Internal state should be taken into account in an approach based on costs and benefits of moving because they represent the justification for moving. They “account for the physiological and, where appropriate, the psychological state of the focal individual, driving the organism to fulfill one or more goals” [14]. In this study, we explicitly modelled two internal states: the stored energy and a representation of the memory of visited locations. Stored energy has indeed been shown to influence herbivores' grazing strategies [13]. We thus computed the cumulative energy over the last 3 days 
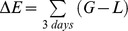
. We also modelled a memory effect, defined as the minimum angle 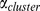 between the step direction and previous clusters of locations, because animals tend to come back to familiar areas for forage and safety [44]. 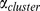 is related to the internal state of the modelled individuals because each modelled individual can remember a limited number of clusters *PatchArray*, characterized by their locations and order of visits. As new clusters are created, if the maximum number of remembered clusters is reached, the oldest ones are deleted, and the list of remembered clusters is updated. If an individual is located in a cluster (*i.e.* less than 1600 m from the cluster's centroid, this state being characterized by a boolean variable *inPatch*), the location of the cluster's centroid is updated based on the animal's movements. For in-depth details on the computation of each variable, and associated submodels and assumptions, we refer the reader to Appendix S1.

To compute these variables, the IBM is made of two entities: individuals, which represent caribou, and the environment. Each of these entities is characterized by state variables (Tables 1 and 2), from which the variables linked to the generative mechanisms were computed.

**Table 1:** State variables for the caribou.

State variables	Unit	Description
*ID*	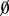	number differentiating the individual from conspecifics
*Location*	(m,m)	the coordinates of the individual location according to the projection UTM NAD 83 Zone19
*Heading*	radians	direction faced by the individual
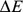	kJ	the energetic balance over the last 72 hours
*inPatch*	Boolean	state of being (1) or not (0) in a patch
*PatchArray*	(m,m, 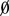 )	array of locations of past patches, and number of locations composing the patch

**Table 2:** State variables for the environment.

State variables	Unit	Description
*Location*	(m,m)	coordinates of the cell according to the projection UTM NAD 83 Zone19
*Altitude*	m	altitude of the cell
*coverType*	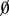	Land-cover type
*Lichen*	grams	quantity of lichens
*Cover*	%	percentage of canopy cover in a 25 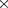 25 m cell
*Edge*	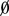	2D gradient of the percentage of canopy cover

### Using a statistical technique as the IBM's trait

#### Process overview and scheduling

The movements of caribou were simulated sequentially by drawing, for each step, 21 random steps from the empirical step length and turning angle distributions. A score was then allocated to each step based on the environmental and state variables related to the generative mechanisms. A step was selected based on these scores and the individual moved to the destination of the step. Individuals then ate, leading to an update of their cumulative energy over the last 3 days 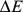, depleting the quantity of lichen *Lichen*, and updating their other internal variables *PatchArray* and *inPatch* based on their new location, and their heading *Heading* based on the performed step (see the description of submodels in Appendix S1 for mathematical details). This process is executed for each individual in series. More details on the variables used in eqn 2 and on the corresponding submodels are provided in section *Parameterization* and in Appendix S1.

Given the number of variables the model simultaneously implements to compute the scores of the steps used in this BCRW, the space of solutions would be too wide to allow for parameterization using an inverse modelling technique such as POM [22], [24], [25]. It would indeed require a large number of patterns [23], and it would also be time consuming as full simulations should be run at each iteration of the parameters' refinement process. This justifies the use of a statistical technique as a forward modelling approach. More specifically, the scores were computed using a Step Selection Function (SSF) [16] based on the variables listed previously, related to the generative mechanisms. SSFs establish the relationship between animal movements and habitat features by comparing observed and random steps. To estimate the SSF, caribou's paths were broken down into steps, which correspond to the straight-line segment between successive locations at 4-hour intervals. Each observed step was then paired with 20 random steps having the same starting point, but differing in length and direction. We used 20 random steps as a compromise between using enough steps to be able to detect all of the possible 15 landcover classes and the practical necessity to keep the computational requirements of the model low. This order of magnitude is consistent with other studies which consider only 10 steps, but also fewer habitat classes, *e.g.*, [45]. The lengths and turning angles of random steps were drawn from the empirical distributions comprised of the steps of all individuals collected during the winter period, from December 28th to April 15th [31]. The characteristics of pairs of observed and random steps were compared with a conditional logistic regression, using the sets of observed and random steps sharing the same starting point as stratums. The resulting SSF took the general form:
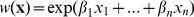
(2)where 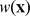 represents the SSF score for the step described by the vector **x** of variables *x_i_* associated with each observed or randomly drawn step, and 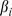 is the coefficient corresponding to *x_i_*. A positive coefficient 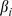 indicates a selection for the variable (the individual is more likely to use a step associated to positive values of *x_i_*), whereas a negative coefficient means that the variable is avoided. For mathematical details on SSF assessment, see Fortin et al. [16] and Forester et al. [46].

#### Keeping track of internal state variables

This approach requires that for each observed and random step, the variables included in the SSF can be computed. Amongst the environmental and state variables related to the generative mechanisms, *L*, *Cover*, *Edge*, 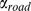, *D_road_*, 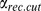, 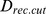, 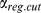, and 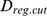 are related to habitat features and can easily be obtained from the cartographic and animal tracking data (see Appendix S1 for the mathematical details to compute these variables). However, this is not true for *G*, 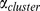, and 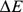. On the one hand, *G* and 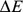 require the use of both an energetic model and a resource depletion model (*G* is a function of the quantity of resources at the end of the step, which is depleted when the animal eats; 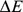 is the sum of *G* – *L* over 3 days, *i.e.* 18 steps). On the other hand, 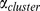 requires a progressive recording and update of successive clusters of locations of the real animals, as animals can come back to an already existing cluster, which then needs to be updated (clusters were created dynamically if consecutive locations were close to each other, and are characterized by their centroid, which is updated if individuals go back to an existing cluster; if the maximum number of clusters is reached and a new cluster is created, the oldest cluster is deleted from the memory; see Appendix S1 for mathematical details).

**Figure 1:**
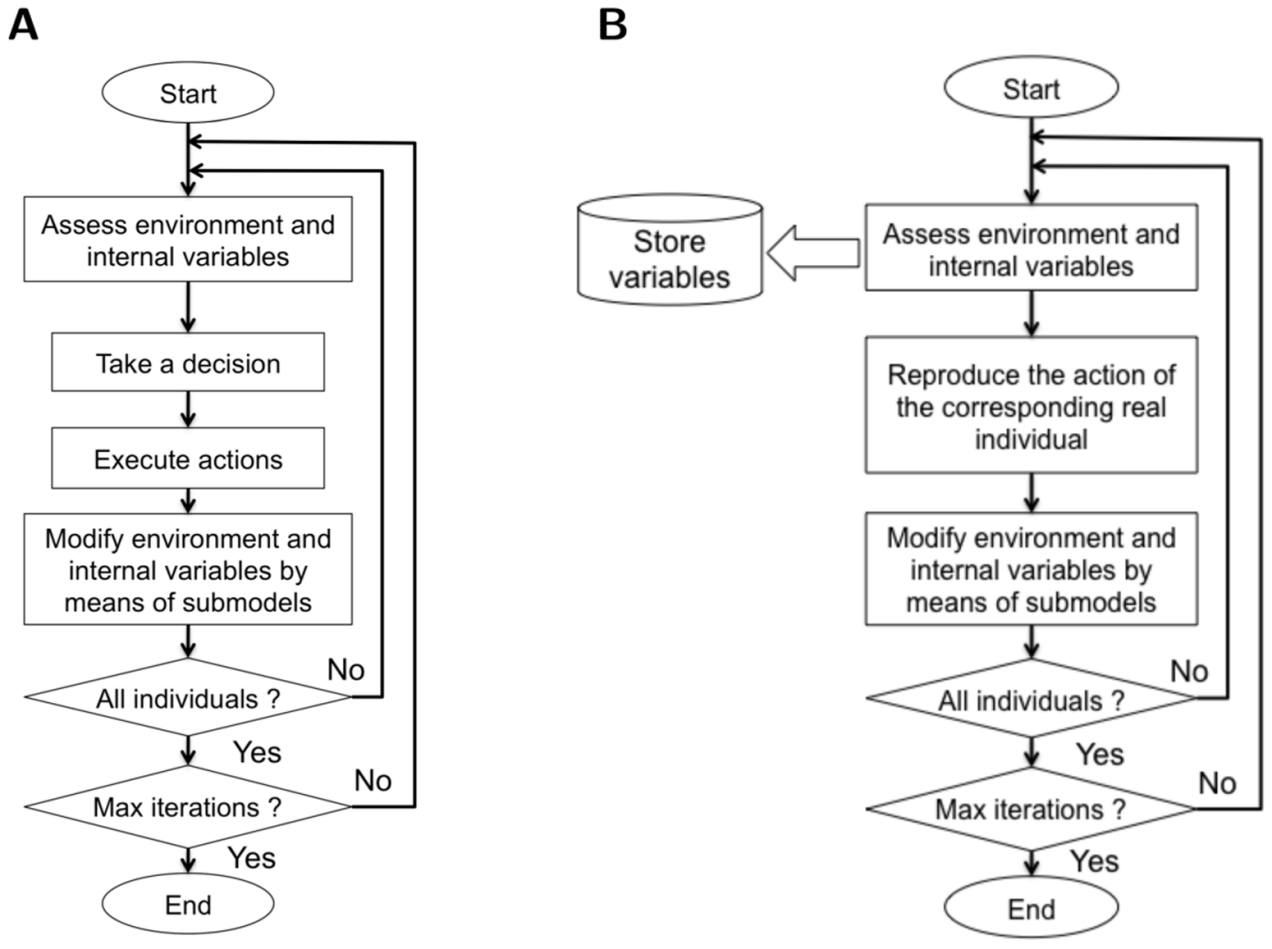
IBM and data generator scheduling. Comparison of schedules of (a) an IBM, and (b) modification of the IBM to generate surrogate data. Rectangles represent an action, while diamonds check if a condition is completed and to decide on the next action according to the result.

To generate these data, we replicated the steps of real individuals (as recorded in the GPS data) in the simulated environment, and simultaneously executed the different submodels described in Appendix S1 to update the internal and environmental variables, as in an IBM. For each observed and replicated step of each animal, we drew 20 random steps, and computed and recorded the variables *G*, *L*, 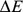 and 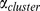 for these steps too (Fig. 1). At each iteration, the values of the different variables were recorded for each observed and random step (note that internal and environmental variables were not updated for random steps) to create a database that allowed for the estimation of the SSF by means of traditional conditional logistic regression.

## Parameterization

### SSF models

To account for trade-offs between costs and benefits of moving, variables related to energy rate, predation risk, and human-induced disturbance were included in eqn 2. Three different SSFs of increasing complexity were fitted using the following models: 1) a memory model which only takes into account spatial memory:
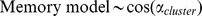



2) an energetic model which also takes into account the ratio between energy gains and expenditures:
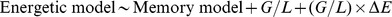



3) an environment model which also takes into account environmental variables and human disturbance:
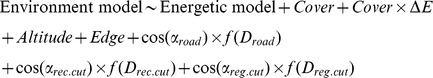



### Statistical details

Because caribou locations were measured every 4 hours, successive steps were not independent. Robust standard errors of SSF parameters can be estimated using a robust sandwich estimate of the covariance matrix [47]. This approach requires partitioning the data into clusters of autocorrelated steps, each cluster being independent from the others [47], [48]. Autocorrelation and partial-autocorrelation analyses of the deviance residuals showed that autocorrelation disappeared beyond lag 2. We thus ensured that all clusters were independent by removing locations so that the last location of a given cluster was separated by at least 2 steps (8 hours) from the first location of the next cluster. To assess whether movements were independent amongst radio-collared individuals, we estimated the distance between simultaneous locations of all individuals. Individuals were considered independent if they were separated by more than 100 m [29]. When two individuals were closer than 100 m, the two clusters to which these locations pertained were merged into a single cluster. Finally, variance inflation factors (VIF) were 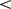
3 for all variables, indicating a lack of multicolinearity [49]. The data used for parameterizing the SSF can be found online in the supplementary material.

### Parameterization results

The level of empirical support received by the SSF was assessed for each model using the quasi-likelihood under independence criterion (QIC) [50]. The QIC accounts for nonindependence between subsequent observations by being calculated while also considering independent clusters of observations [51]. Like the Akaike Information Criterion (AIC), the QIC penalizes over-complexity by adding a penalty term for the number of parameters, thus allowing for a compromise between parsimony and fitting capacities, to prevent overfitting. The Environment model including all memory, energetic and environment variables explained the movement data better than the simpler models, meaning that the latent covariables were relevant (Table 3). Caribou biased their movements towards previously visited patches (Table 4). They selected steps with a high ratio of energy gains over energy expenditures. They selected areas of low canopy cover, of high altitude, and avoided roads and regenerating cuts. When their energy level over the last 3 days was high, they increased their selection of areas of low canopy cover.

**Table 3:** Model selection amongst the candidate models of step selection by woodland caribou in the Côte-Nord region, Québec (Canada), in winter.

No.	Candidate Model	*K*	QIC	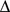 QIC	*w_i_*
1	Mechanistic model	1	144632.3	546.6	0%
2	Energetic model	3	144197.3	111.6	0%
3	Environment model	10	144085.7	0	100%

Number of parameters (K), QIC scores, differences in QIC compared to lowest scoring model (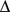 QIC) and QIC weights (*w_i_*) for the three candidate models.

**Table 4:** Coefficients (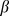), standard error (SE) and 95% confidence intervals (CI) for the complete SSF model for the woodland caribou in the Côte-Nord region, Québec (Canada), in winter (values are 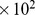).

Variable	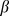	SE	95% CI
cos( 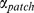 )	37.88	1.48	(34.98∶40.79)*
G/L	14.97	1.70	(11.63∶18.30)*
Cover	−0.16	0.05	(−0.26∶−0.05)*
Altitude	0.23	0.03	(0.18∶0.29)*
Edge	0.02	0.02	(−0.03∶0.06)
cos( 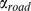 ) 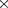 f(D*_road_*)	−9.59	4.47	(−18.35∶−0.83)*
cos( 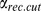 ) 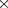 f(D 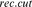 )	0.21	9.83	(−19.05∶19.48)
cos( 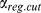 ) 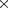 f(D 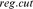 )	−62.99	21.04	(−104.23∶−21.77)*
G/L 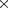 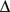 E	16.25	2.90	(10.58∶21.93)*
Cover 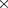 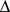 E	−0.34	0.12	(−0.57∶−0.10)*

*Coefficients for which the 95% confidence intervals excluded zero.

Note that the 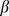-value associated with the term 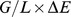 is positive, meaning that the higher the energy balance over the last three days, the more individuals increase the Gain/Loss ratio, which seems counter-intuitive. This is probably due to the fact that a high 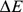 means that the individual is in a patch of high resources, which attracts the individual, and then leads to the formation of a cluster. Another possible explanation of this counter-intuitive result is the fact that in the model, individuals only gain energy at the end of a step. We used this simplifying assumption for computational reasons to avoid having to compute the resource consumption and depletion over all cells along all possible steps. In the IBM, leaving a cluster is thus a combination of a stochastic process, through the generation of random steps and probabilistic selection of the possible steps, and the process of resource depletion.

## Validation

### K-fold cross-validation

We used 5-fold cross-validation for case-control design to evaluate model robustness. An SSF was built using 80% of randomly selected strata. This SSF was then used to estimate 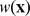 scores for the observed and random steps of the 20% withheld strata. The observed steps of each stratum was ranked against its associated random steps from 1 to 21 (i.e., 21 potential ranks given that a stratum included 1 observed and 20 random locations) based on the 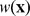 scores, where 1 was the lowest and 21 was the highest possible rank for that stratum. Ranks of observed steps were then tallied into the 21 potential bins. Spearman rank correlation (*r_s_*) was performed between the bin's ranking (121) and its associated frequency. The process was done 100 times, and the average *r_s_* and associated 95% confidence intervals are reported. Model robustness was strong, as indicated by the distribution of observed *r_s_* (0.94, 95% CI: 0.88–0.98), which was higher than expected by chance alone (−0.02, 95% CI: −0.49–0.40).

### Model validation based on emergent patterns

An additional (and more common) means to validate the IBM is to verify that the generative mechanisms used as a trait of the model allows for the reproduction of patterns at higher levels of emergence. By doing so, we verify that our understanding of the system's mechanisms is robust, and according to the level of emergence paradigm, this should ensure the generalization ability of the IBM. Contrary to k-fold cross-validation, this process can thus be characterized as a *vertical* process.

**Figure 2:**
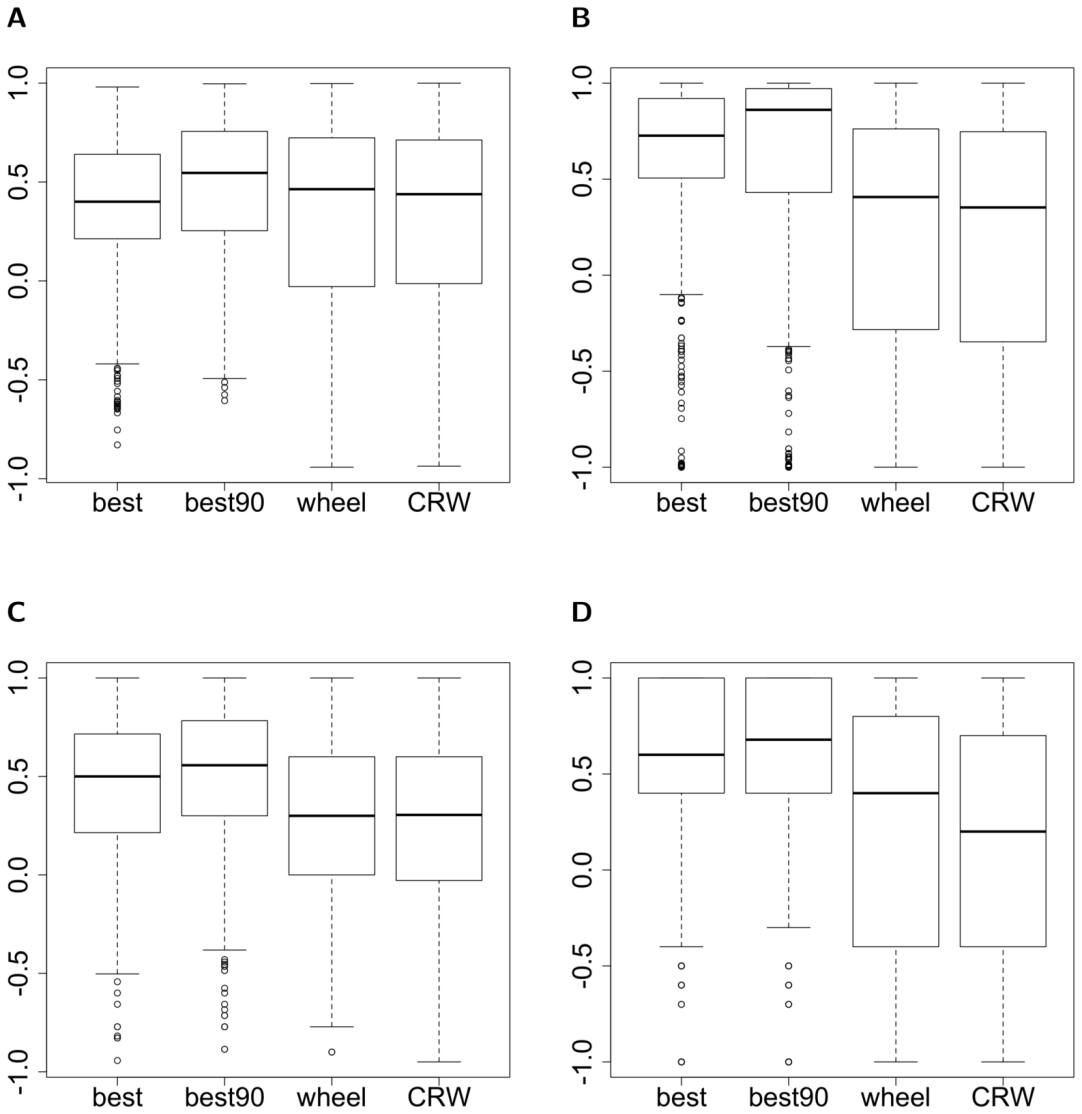
Validation of the habitat selection pattern. Distribution of the Pearson *R* and Spearman *r_s_* correlation coefficients between the 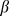-value vectors for real and simulated individuals for the IBM with the different step selection methods based on the SSF scores of the steps (the *best*, the 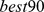 and the *roulette wheel* methods) and a simple CRW (which selects any step with equal probability): (a) *R* is computed over all 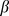-values, (b) *R* is computed over 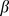-values whose 95% confidence intervals exclude 0, (c) *r_s_* is computed over all 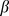-values, and (d) *r_s_* is computed over 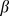-values whose 95% confidence intervals exclude 0. A high correlation coefficient means that simulated individuals select land cover types in a similar fashion to real individuals, hence validating the model. A generalized linear mixed model showed that the *best* and 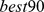 methods performed better than the 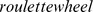 method and the CRW (*R* on all 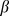-values: *best*, t = 1.79, p-value
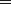
0.0738 - 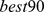, t = 5.72, p-value
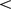
0.0001 - *CRW*, t = −0.55, p-value
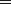
0.5836; *R* on 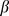-values whose 95% confidence intervals excluded 0: *best*, t = 11.33, p-value
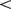
0.0001 - 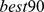, t = 12.55, p-value
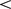
0.0001 - *CRW*, t = −0.76, p-value
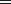
0.4492; *r_s_* on all 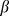-values: *best*, t = 9.12, p-value
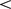
0.0001 - 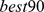, t = 11.80, p-value
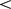
0.0001 - *CRW*, t = −0.56, p-value
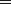
0.5782; *r_s_* on 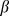-values whose 95% confidence intervals excluded 0: *best*, t = 11.02, p-value
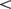
0.0001 - 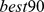, t = 11.66, p-value
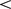
0.0001 - *CRW*, t = −1.35, p-value
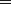
0.1757).

First, we verified that modelled individuals would select the different landcover types similarly to their real counterparts. We simulated as many individuals as were represented in the GPS data for each year, for 648 iterations (corresponding to the 108 days of the winter season, multiplied by 6 steps per day) and initialized their locations at the same coordinates. Simulated individual movements were confined to the home ranges of the real individuals by deleting and re-drawing any random step falling outside of the home range of the corresponding real individual, to remove the influence of differences of home range between real and simulated individuals on land-cover types selection. We tested 4 different selection methods for the steps based on their score: the *best* method, when the step with the highest score is always selected, the *best90* method, when the step with the highest score is selected 90% of the time, and any step has an equal probability to be selected the remaining 10% of the time, the *roulette wheel* method, in which each step has a probability 
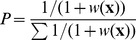
 to be selected (the logistic function was used instead of the raw score because it represents the probability of use conditioned on habitat [52]), and a simple CRW method in which every step has an equal probability of being chosen, which represents the null model. The model was then run 20 times for each year and each selection method. For each model, we estimated Resource Selection Functions (RSF [53]) on landcover types for each real individual and for each replication of the simulated individuals [30]. RSF compare locations of individuals with random locations drawn, in our case, in the home range of the individuals (defined here as the 95% minimum convex polygon), and use logistic regression by a generalized linear mixed model (GLMM, using a logit function as the link function) to estimate the model's coefficients (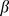), following the same principle as an SSF. Each RSF produced a vector of 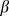-values representing habitat selection. Patterns of habitat selection by separate individuals were then compared for each selection method by computing a Pearson *R* and a Spearman's *r_s_* correlation coefficient computed between the vector of 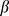-values of a real individual and the vectors of the 20 runs of the corresponding simulated individual, for all 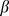-values and for 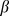-values whose 95% confidence intervals excluded 0. For each year, this procedure produced 20 correlation coefficients for each monitored individual. We compared the four models using generalized linear mixed models using the individuals and the years as random effects, to explain the proportion of explain variance. Results show that the *best* and 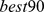 methods performed significantly better than the 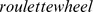 method and the CRW (*R* on all 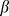-values: *best*, t = 1.79, p-value
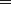
0.0738 - 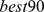, t = 5.72, p-value
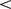
0.0001 - *CRW*, t = −0.55, p-value
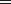
0.5836; *R* on 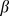-values whose 95% confidence intervals excluded 0: *best*, t = 11.33, p-value
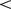
0.0001 - 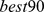, t = 12.55, p-value
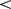
0.0001 - *CRW*, t = −0.76, p-value
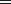
0.4492; *r_s_* on all 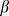-values: *best*, t = 9.12, p-value
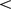
0.0001 - 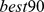, t = 11.80, p-value
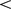
0.0001 - *CRW*, t = −0.56, p-value
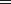
0.5782; *r_s_* on 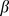-values whose 95% confidence intervals excluded 0: *best*, t = 11.02, p-value
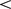
0.0001 - 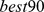, t = 11.66, p-value
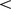
0.0001 - *CRW*, t = −1.35, p-value
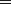
0.1757; Fig. 2).

**Figure 3:**
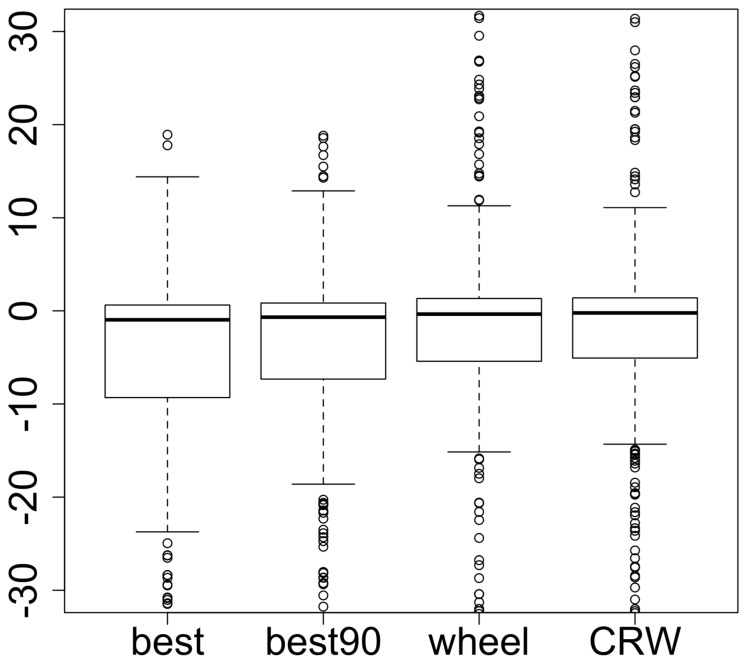
Validation of the home range size pattern. Distributions of the 95% minimum convex polygons areas for the different step selection methods based on the SSF scores of the steps (the *best*, the 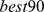 and the *roulette wheel* methods) and a simple CRW (which selects any step with equal probability). The *best* and 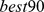 methods produced slightly smaller home ranges than the *roulette wheel* one and the CRW (Kruskal-Wallis test: 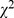 = 26.72, df = 3, p-value
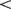
0.0001).

We then verified that the size of simulated and real individuals' home ranges would also coincide. We simulated as many individuals as represented in the GPS data for each year, and initialized their locations at the same coordinates, but this time without restraining their movements. We then compared the areas of the 95% minimum convex polygons (MCP) for the locations of each real and simulated individual for each year and for each selection method. To do so, we divided the simulated areas by the real ones when simulated areas were higher, and we divided the real areas by the simulated ones 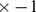 when simulated areas were smaller. This results in a coefficient 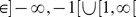. We therefore added and subtracted 1 to the negative and positive values, respectively, to obtain a coefficient 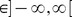, for which 0 means that home ranges have the same area. The *best* and 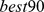 methods produced slightly smaller home ranges than the *roulette wheel* one and the CRW (Kruskal-Wallis test: 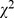 = 26.72, df = 3, p-value
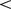
0.0001), and smaller than observed in the real system, but the simulated values of our index still encompass 0 (Fig. 3). Simple CRWs should not allow to model persistent home ranges on the long term [54]. The fact that the CRW produces realistic home ranges in this case means that the length of the winter season is too short to allow for the ability of the model to produce a home range to be truly observable. However, the fact that the *best* and *best90* methods show a tendency to limit the displacements of simulated individuals to smaller areas suggests that the memory effect represented by the 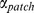 variable allows for the establishment of a home range, and shows the potential of the model to maintain the home range pattern when simulating longer periods.

## Simulations of environmental changes

To illustrate the ability of the IBM to simulate the behavioural responses of individuals to environmental changes, we ran simulations for different environments, using the 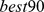 selection method. We divided the simulated area into 225 km^2^ quadrats (corresponding to the current size of forest harvesting blocks), and changed the landcover types at the center of each quadrat other than fixed open areas and water bodies to mixed forest stands to represent a phenomenon of encroachment of deciduous trees, which is a common consequence of forest harvesting. This resulted in modifying the amount of resources, percentage of canopy cover, etc. for woodland caribou, because they consume preferably terrestrial lichens in winter [39]. We tested 2 different environments. In the first one, we used the 2009 environment for the study area without modifying the landcover types. In the second one, we replaced the landcover types in a square representing 70% of each quadrat by mixed forest, which corresponds to the maximum level of harvest permitted under current logging practices.

**Figure 4:**
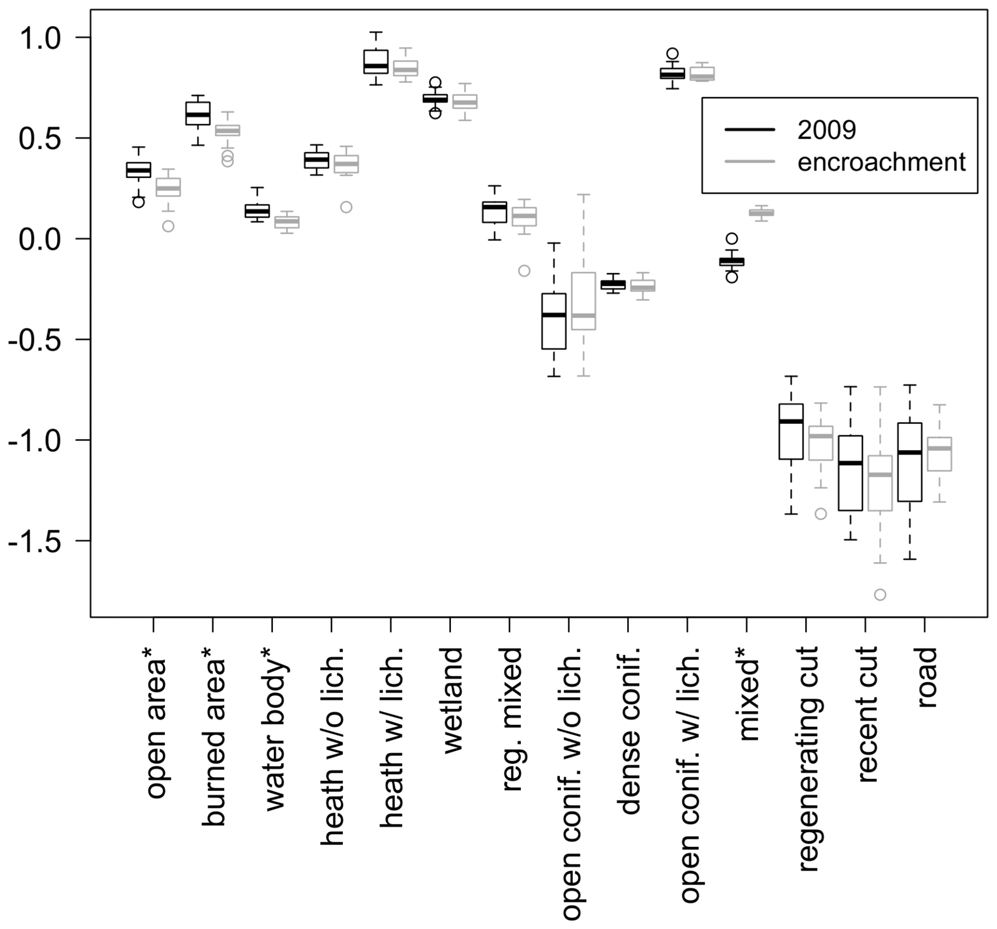
Patterns of habitat selection for the two scenarios. Distributions of the 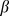-values for the 15 landcover types: 1 = fixed open areas, 2 = burned area, 3 = water, 4 = heath without lichen, 5 = heath with lichen, 6 = wetlands, 7 = regenerating mixed forest, 8 = regenerating coniferous stand, 9 = open conifer stand without lichen, 10 = dense mature conifer forest, 11 = open conifer stand with lichen, 12 = mixed/deciduous forest, 13 = regenerating cut, 14 = recent cut, 15 = road. Open conifer stand without lichen is the class of reference and does not appear in the graph. Asterisks indicate landcover types for which a Mann-Whitney test between distributions produced a p-value
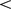
0.05.

For each scenario, we ran 20 simulations with 600 individuals initialized randomly over the open conifer with lichen forest stands, because this landcover type is the species' preferred habitat during winter [30]. During simulations, we recorded the ID, coordinates, and landcover types of all the locations for all individuals. To assess the differences in the selection of the different landcover types with respect to different availability, we computed, for each simulation run, an RSF model over all the individuals, with random points drawn over the whole study area, taking the form: 
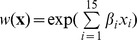
, where *x_i_* is a boolean variable of presence/absence of the corresponding landcover type at the location of the observed of random point. We represented the distributions of each 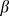-value over the 20 simulations for both scenarios, and verified if the difference of distributions between the two scenarios for each 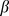-value was significant using a Wilcoxon-Mann-Whitney test for each landcover type (Fig. 4). In response to the encroachment of deciduous trees, individuals decreased their selection of fixed open areas, burned areas and water, but selected mixed and deciduous forests, which were avoided during the 2009 scenario. Other changes in habitat selection were not significant.

**Figure 5:**
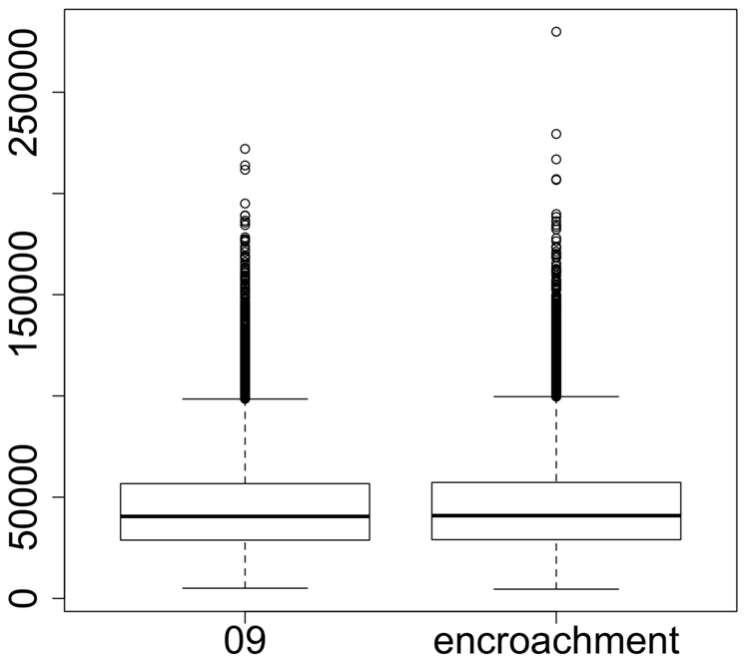
Patterns of home range size for the two scenarios. Distributions of the 95% minimum convex polygons areas for the 2009 and the hardwood encroachment scenarios. A Wilcoxon-Mann-Whitney test showed that distributions were not significantly different (W = 135317637, p-value = 0.22).

To assess the differences in home range sizes, we computed the 95% MCP area of each individual for each replication, and compared the resulting distributions between the two scenarios (Fig. 5). A Wilcoxon-Mann-Whitney test showed that distributions were not significantly different (W = 135317637, p-value = 0.22).

## Discussion

### Model robustness

We designed an individual-based model of woodland caribou's movements with a wide domain of applicability, which can therefore inform on the impact of changes in the composition of the boreal forest's landscape due to management plans and other human and natural disturbances, on caribou habitat selection. To ensure that the model could be applied to a broad range of environmental conditions which have not yet been observed in the real system, we used generative mechanisms at a low level of emergence based on internal state variables (energetic requirements and spatial memory) and habitat features as a trait. Movements were modelled by an SSF calibrated using conditional logistic regression, *i.e.* forward modelling. SSF calibration was made possible by the generation of internal state variables using an IBM-based framework. Contrary to an inverse modelling process, the SSF calibration involved model selection using information theory, which allows for the statistical dismissal of over-complex models by penalizing the model's performance with the number of parameters. Note that, due to this model selection process, forward modelling also provided the advantage over pure inverse modelling of being faster, as it did not require to iteratively run simulations to adjust the IBM parameters, which can be extremely computationally intensive.

If the parameterization process allows for the selection of the necessary variables, the purpose of validation is to ensure that the variables are also sufficient to explain and reproduce the emergent patterns, related in our case to the animals' movements. IBMs are usually validated by reproducing patterns of comportments at higher levels of emergence, which we performed using patterns of habitat selection and spatial distribution. This is thus a vertical validation, which ensured that the model captured the processes of the natural system. As a general rule, the strength of the validation procedure depends on the number of comportments which can be compared. In this work, we applied vertical validation with respect to only two comportments, namely habitat selection and home range size.

When only few comportments can be used for vertical validation, using statistical techniques such as SSFs for the forward modelling of the IBM trait provides the advantage of allowing for additional horizontal validation, *i.e.* using data from variables used in the IBM's trait, using k-fold cross-validation. Like any cross-validation process, k-fold cross-validation proceeds by reducing the size of the training base and then verifies that the rest of the data are correctly explained by the SSF assessed on this reduced database. The subtlety lies in the fact that the process is repeated a certain number of times, randomizing the data partition, to account for the small size of the database. Because generative mechanisms should be able to explain the data whatever the range of environmental conditions, k-fold cross-validation is therefore a relevant method to verify the fact that the variables used in an SSF model allow for generalization to different environments. Because it makes use of data at the same level of emergence, k-fold cross-validation can be characterized as a *horizontal* process (*c.f.* Fig. 4 of [23]). One drawback of k-fold cross-validation is that its capacity to assess the generalization ability of the IBM depends on the variability of environmental conditions in the database. If the environment is uniform, the environmental conditions represented by the remaining data will be similar to the conditions used to assess the SSF, and results are therefore expected to be conclusive. Each approach thus has its own limitations, but using a combination of horizontal k-fold cross-validation and vertical validation based on emergent patterns increased our confidence in the robustness of the model.

Important changes in the composition of the environment are likely to generate a functional response in the selection of the landcover types by caribou. This phenomenon has been observed for local changes in the environment, *e.g.*, [55]. However, functional responses are hard to predict, both qualitatively and quantitatively, because they emerge from generative mechanisms at a lower level of emergence. This is especially true at large spatial and temporal scales because of the difficulty to obtain data at such scales. On the other hand, studies based on local changes in the environment may not encompass the global changes that may occur over long periods and large areas, potentially making management plans based on such limited observations ill-adapted in a conservation or management context. The processes of the IBM presented in this study were based on trade-offs between costs and benefits of moving, i.e. generative mechanisms at a low level of emergence, which allowed the IBM to produce an emergent functional response in the selection of landcover types by simulated caribou when the landscape was subject to a large-scale change in the environment, in this case the extreme encroachment of deciduous trees. This functional response can also be quantified, hence highlighting the potential for such a model in a conservation context. Moreover, the model allowed us to assess the functional response at the level of a population and at the scale of the landscape, whereas empirical studies usually assess it for separate individuals and at smaller spatial scales (*e.g.* inter home-range level [35], [56]–[58] or within home-range level [55]), because of the limitations resulting from a restricted number of monitored individuals.

### Ecological relevance

Increasing the amount of mixed and deciduous stands in the landscape produced two types of response from simulated individuals. First, they decreased their selection of landcover types with a low amount of resources and low canopy cover. This may be due to the fact that, according to the SSF that serves as a trait (Table 4), individuals decrease their selection of landcover types having low canopy cover, which provide a low ratio between energy gains and expenditures as their energy level decreases. Because mixed and deciduous forests do not contain a lot of resources for caribou, their energy level was likely to be lower for the scenario of encroachment of deciduous trees than for the 2009 landscape. Simulated individuals also selected mixed and deciduous forest stands in the encroachment of deciduous trees scenario, while they avoided them in the 2009 landscape. This is probably due to the fact that they have to travel more through this landcover type to access resources, given its widely spread spatial configuration. Because woodland caribou in the study area are part of a predator-prey network involving multiple species at different trophic levels, this functional response can have important repercussions on caribou populations. Encroachment of deciduous trees in the study area is likely to result in an increase of moose (*Alces alces*, G.) abundance, and therefore of gray wolf (*Canis lupus*, L.) density, as moose is its main prey [59]. Consequently, this may in turn increase the predation rate of wolves on caribou, a phenomenon known as apparent competition [60]. Moreover, wolves select mixed and deciduous forest stands in winter in the study area, probably because it is the preferred landcover types of moose [30]. As such, the increased predation rate on caribou that may result from the encroachment of deciduous trees through apparent competition would probably be intensified due to the selection of mixed and deciduous forest stands by caribou. Simulating moose and wolves in addition to caribou would be required to confirm this hypothesis.

## Conclusions

In this study, we designed an individual-based model of caribou's movements based on generative mechanisms at low levels of emergence to ensure its generalization ability. We used a statistical technique to parameterize the IBM's trait according to forward modelling principles. This was made possible by the artificial generation of surrogate data for the variables at low levels of emergence which were not directly accessible from the GPS data.

This procedure ensured that the trait of the IBM was statistically relevant to explain the data, but also that the parameters identified by the statistical modelling allowed for the generation of behaviours at higher levels of emergence. These two aspects demonstrated both the necessity and sufficiency of the variables used in the SSF model to explain the system's behaviour of interest. Using a statistical model as a trait of the IBM also increased its robustness because it allowed to use two independent validation processes: k-fold cross-validation, a *horizontal* process which uses data at the same level of emergence as the data used for parameterization, and emergent patterns validation, a *vertical* process which ensures that the model allows for the reproduction of patterns at higher levels of emergence. Moreover, by favouring forward modelling, this method also has the advantage of reducing the number of iterations that would be required by pure inverse modelling methods.

Generating functional responses suggests that our framework permitted to design a model that encompasses some aspect of the complexity of the system, and, thus, that the approach is adequate to provide projections on future possible states of the system in reaction to different management plans. This is especially relevant to test the long-term impact of scenarios corresponding to environmental configurations that have yet to be observed in the real system.

## Supporting Information

Appendix S1
**Submodels.** Mathematical details and assumptions of the submodels for the computation of the generative mechanism-related variables. **Figure S1.** Relationship between the number of clusters and the number of caribou locations. For each individual for each year, we took the first 100 locations, then the first 200 locations, and so on until the total number of locations, and we plotted the number of clusters versus the number of locations, which is represented by the circles. The solid line represents the median of the number of clusters over all individuals for each hundred locations, and the error bars show the lower and upper quartiles. **Figure S2.** Impact of anthropogenic features on movement. Changes in the cosinus of mean orientation for real steps with respect to (a) the nearest road, (b) recent cut, and (c) regenerating cut, as a function of distance from these anthropogenic features for 22 radio-collared caribou during winter in the Côte-Nord region of Québec, Canada. For example, caribou traveling perpendicular to and leading away from the nearest disturbed area were assigned −180*°* (producing a cosinus value of −1), whereas those travelling directly towards the area were assigned 0*°* (producing a cosinus value of +1). For each 100 m interval, we then computed the mean of the orientation over all locations and all individuals. The left axis indicates the mean value for points taken from the GPS data. The relation between the distance from anthropic perturbations and the angle was approximated as linear at first, and to disappear after some distance, as shown by the solid line. The distance at which the influence of distance was considered to disappear (and at which the linear function crosses 0) corresponds to the first point superior or equal to 0, *i.e.* 1500, 1600 and 1300 m for roads, recent cuts and regenerating cuts, respectively. The linear function 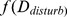 was scaled between 0 and 1 in order to give more influence to the direction of a step when close to a disturbance (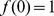), and no influence when the distance is high, hence the inverse right axis.(PDF)

Data S1 **Supporting data.**
(ZIP)
