# Supplementary material for: Uniting Statistical and Individual-Based Approaches for Animal Movement Modelling
Source: PLoS One. 2014 Jun 30;9(6):e99938. doi: 10.1371/journal.pone.0099938 (PMC4076191; doi:10.1371/journal.pone.0099938)
Supplement: Appendix S1 — Submodels. Mathematical details and assumptions of the submodels for the computation of the generative mechanism-related variables. Figure S1. Relationship between the number of clusters and the number of caribou locations. For each individual for each year, we took the first 100 locations, then the first 200 locations, and so on until the total number of locations, and we plotted the number of clusters versus the number of locations, which is represented by the circles. The solid line represents the median of the number of clusters over all individuals for each hundred locations, and the error bars show the lower and upper quartiles. Figure S2. Impact of anthropogenic features on movement. Changes in the cosinus of mean orientation for real steps with respect to (a) the nearest road, (b) recent cut, and (c) regenerating cut, as a function of distance from these anthropogenic features for 22 radio-collared caribou during winter in the Côte-Nord region of Québec, Canada. For example, caribou traveling perpendicular to and leading away from the nearest disturbed area were assigned −180° (producing a cosinus value of −1), whereas those travelling directly towards the area were assigned 0° (producing a cosinus value of +1). For each 100 m interval, we then computed the mean of the orientation over all locations and all individuals. The left axis indicates the mean value for points taken from the GPS data. The relation between the distance from anthropic perturbations and the angle was approximated as linear at first, and to disappear after some distance, as shown by the solid line. The distance at which the influence of distance was considered to disappear (and at which the linear function crosses 0) corresponds to the first point superior or equal to 0, i.e. 1500, 1600 and 1300 m for roads, recent cuts and regenerating cuts, respectively. The linear function was scaled between 0 and 1 in order to give more influence to the direction of a step when close to a disturbanc [file pone.0099938.s001.pdf]

Appendix S1: Submodels

Caribou

Energy models

Energy gains (G) Caribou behaviour is influenced by forage distribution [1]. To compute the expected potential gain at the end of a step, a Michaelis-Menten equation (1) was used to model the short-term functional response of lichen consumption X [2].

$$X(V) = \frac{aV}{b + V} \tag{1}$$

where a is the maximum rate of consumption, b is the foraging efficiency, *i.e.* the resource biomass for which intake is one-half of the maximum rate, and V is the resource biomass of the cell. a was set to 61.3 g/day per kg body weight [3]. b was set to 40 g/m^2 [2]. V varied according to the land cover type, and was taken from [4] (see Section Environment hereafter for details on the amount of resources). X was then converted into energy gains, with a rate of 7.79 kJ/g (1.86 kcal/g) [3], in order to be more easily compared with energy expenses due to displacements.

Energy expenses (L) For each 4-hour step, energy expenditures are computed by summing the basal metabolic rate for a time step, the energy expenses due to the travelled distance $Dist$ (in km), and the energy expenses due to the difference of altitude $Elev$ (in km) along a step [5, 6]:

$$\mathrm{L} = \begin{cases} 431.5 \times M^{0.75}/6 + 2.64 \times M \times Dist + 31 \times M \times Elev, & \text{if } Elev \geq 0 \\ 431.5 \times M^{0.75}/6 + 2.64 \times M \times Dist - 5.7 \times M \times Elev, & \text{if } Elev < 0 \end{cases} \tag{2}$$

where M is the mean body mass of a caribou, in kg. The weights of the caribou in the studied population were not available. We thus estimated the mean body mass to be 147 kg, based on studies in Alberta [7].

Spatial memory: recording and updating clusters of locations

In the IBM, the home range should emerge from the structure of the landscape, and cannot be set *a priori* by means, for example, of a central attraction point. Van Moorter et al. [8] showed home ranges can emerge from a BCRW approach, when movements are biased towards previously visited patches. Each visited patch was recorded and characterized by a reference and a working memory, based on its intrinsic utility value, and on how recent the last visit was, respectively. These two memory values were then used to allocate a value to a patch, used to bias movements. The ability of animals to remember patches and go back to them, and the home range emerging from this ability is usually explained by the fact that it should provide some familiarity with the environment, and therefore increase safety with respect to food resources and predation risk [9].

In our model, each caribou has the ability to remember suitable patches it has visited. Patches were defined as clusters of individual's locations, *i.e.* a limited area where an individual tends to spend a significant amount of time. This was used instead of, for example, predefined patches of food based on land cover types, because the definition of a suitable patch is more complex, and results from a trade-off between forage requirements and shelter from predation risk, provided by landscape characteristics [10]. A cluster may therefore result from a combination of different characteristics of the landscape in a given area, and the history of the individual, because it defines its internal variables.

Clusters of individual's locations should serve as an indicator of the patches they consider as suitable in the landscape, and these clusters should emerge in the final IBM. This approach has the advantage that one does not need to define *a priori* what represents a suitable patch, which could depend on different parameters (landscape characteristics, size of the patch, etc.), and on the internal state of the individual.

In the IBDG and in the IBM, if an individual has not visited any patch yet, a new temporary patch is created if 2 consecutive steps are separated by less than 1600 m. Each cluster is then characterized by the location of its center of gravity computed over all the steps taken in the cluster. It is considered that the individual remains in the same cluster as long as it does not move from the center of gravity by more than 1600 m. After each step in a patch, the center of gravity location of the patch is updated, and the number of steps made in a given patch is incremented. Once an individual leaves a cluster, the cluster's center of gravity is added to the individual's memory.

To determine this distance of 1600m, we used a nearest neighbour hierarchical clustering algorithm to group caribou locations from the GPS data for each individual and for every year. If caribou go back

to previously visited clusters, the number of clusters should not be proportional to the total number of locations, but we should observe a plateau of the number of clusters as the total number of locations increases. For each individual for each year, we took the first 100 locations, then the first 200 locations, and so on until reaching the total number of locations recorded for that individual, and we plotted the number of clusters versus the number of locations. We repeated this operation for thresholds from 600 to 2500 m, with an increment of 100 m. When plotting the median of the number of clusters for each hundred of locations, a threshold of 1600 m allows to observe the clearest plateau (Fig. S1).

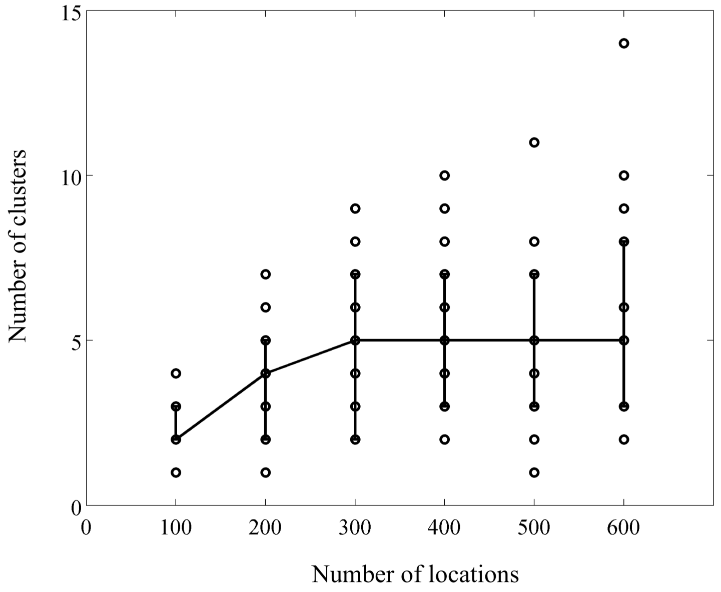

Figure S1. Relationship between the number of clusters and the number of caribou locations.
For each individual for each year, we took the first 100 locations, then the first 200 locations, and so on until the total number of locations, and we plotted the number of clusters versus the number of locations, which is represented by the circles. The solid line represents the median of the number of clusters over all individuals for each hundred locations, and the error bars show the lower and upper quartiles.

Reaction to anthropogenic disturbance

Measuring the orientation of caribou steps with respect to anthropogenic disturbance shows that caribou move more perpendicularly to anthropogenic disturbance directly at the disturbance, and tend towards randomness when moving away from it (Fig. S2). This aspect of animal behaviour was incorporated in the model by including the following term in the SSF model for each anthropogenic disturbance (namely

roads, recent cuts and regenerating cuts):

$$\cos(\alpha_{disturb}) * f(\mathrm{D}_{disturb})$$

where $\alpha_{disturb}$ is the orientation of the individual with respect to the nearest anthropogenic feature, and $f(\mathrm{D}_{disturb})$ is a coefficient bounded between 0 and 1, depending on the distance to the nearest anthropogenic disturbance. By using discounting functions, we give more importance to the angle when the distance is small, and no importance when it is great. Eqn 3, 4, and 5 were used for roads, recent cuts, and regenerating cuts, respectively. Each equation corresponds to a linear function that crosses the y-axis for the first value when the cosinus of the mean of the angle was inferior or equal to 0, *i.e.* 1500 m, 1600 m, and 1300 m for roads, recent cuts, and regenerating cuts, respectively, and that crossed the x-axis when the cosinus of the mean of the angle was superior or equal to 0 . We then took the absolute value of this function and scaled it between 0 and 1 (Fig. S2).

$$f(D_{road}) = \begin{cases} -\mathrm{D}_{road}/1500 + 1, & \text{if } D_{road} < 1500 \\ 0, & \text{if } D_{road} \geq 1500 \end{cases} \tag{3}$$

$$f(D_{rec.cut}) = \begin{cases} -\mathrm{D}_{rec.cut}/1600 + 1, & \text{if } D_{rec.cut} < 1600 \\ 0, & \text{if } D_{rec.cut} \geq 1600 \end{cases} \tag{4}$$

$$f(D_{reg.cut}) = \begin{cases} -\mathrm{D}_{reg.cut}/1300 + 1, & \text{if } D_{reg.cut} < 1300 \\ 0, & \text{if } D_{reg.cut} \geq 1300 \end{cases} \tag{5}$$

Environment

Resources

Resource quantities V were estimated from the literature. We used Courtois [4]'s estimates of lichen biomass in different land-cover types, as follows: fixed open areas = 500 kg / ha, burned area = 0 kg / ha, water = 0 kg / ha, heath without lichen = 930 kg / ha, heath with lichen = 3895 kg / ha, wetlands = 500 kg / ha, regenerating mixed forest = 225 kg / ha, regenerating coniferous stand = 484 kg / ha,

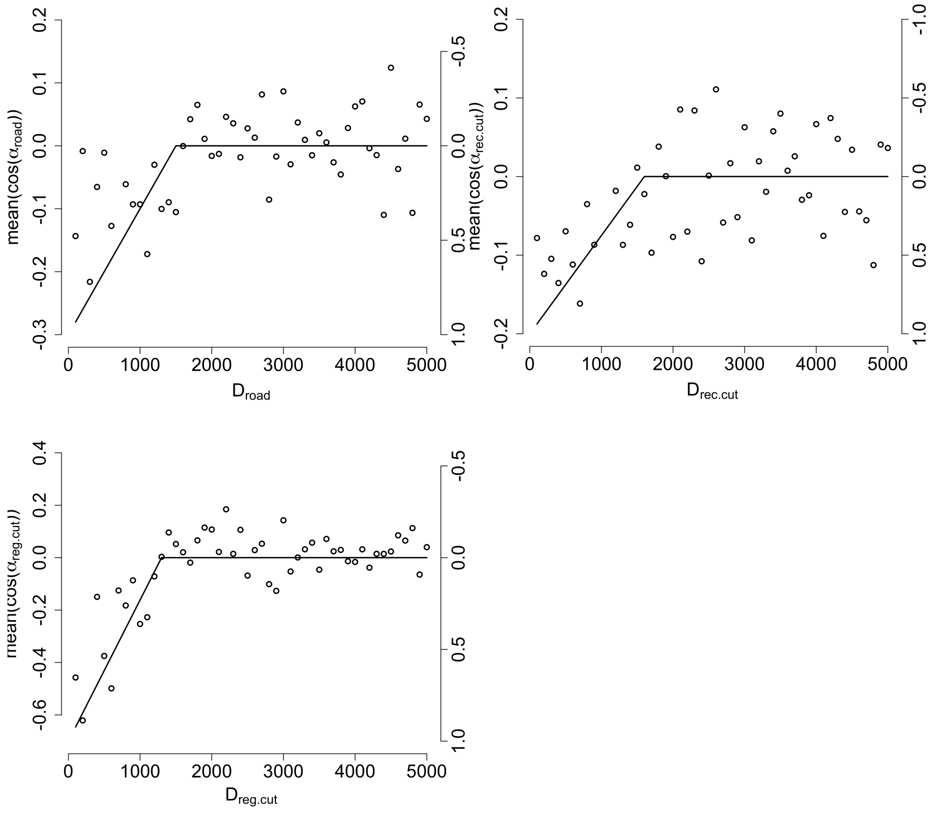

Figure S2. Impact of anthropogenic features on movement.
Changes in the cosinus of mean orientation for real steps with respect to (a) the nearest road, (b) recent cut, and (c) regenerating cut, as a function of distance from these anthropogenic features for 22 radio-collared caribou during winter in the Cte-Nord region of Qubec, Canada. For example, caribou traveling perpendicular to and leading away from the nearest disturbed area were assigned -180° (producing a cosinus value of -1), whereas those travelling directly towards the area were assigned 0° (producing a cosinus value of +1). For each 100 m interval, we then computed the mean of the orientation over all locations and all individuals. The left axis indicates the mean value for points taken from the GPS data. The relation between the distance from anthropic perturbations and the angle was approximated as linear at first, and to disappear after some distance, as shown by the solid line. The distance at which the influence of distance was considered to disappear (and at which the linear function crosses 0) corresponds to the first point superior or equal to 0, *i.e.* 1500, 1600 and 1300 m for roads, recent cuts and regenerating cuts, respectively. The linear function $f(D_{disturb)}$ was scaled between 0 and 1 in order to give more influence to the direction of a step when close to a disturbance ($f(0) = 1$), and no influence when the distance is high, hence the inverse right axis.

open conifer stand without lichen = 312 kg / ha, dense mature conifer forest = 312 kg / ha, open conifer stand with lichen = 2575 kg / ha, mixed/deciduous forest = 31 kg / ha, regenerating cut = 0 kg / ha, recent cut = 0 kg / ha, road = 0 kg / ha.

Resource depletion was modelled by means of the resource consumption model presented previously. Because caribou consume terrestrial lichen, they destroy about ten times more lichen than they consume [4], and resources were therefore updated by $V = V - 11 * X(V) \times (4hours)$.

Cover and Edge

The percentage of canopy cover was included into the Landsat Thematic Mapper images class descriptions. The Edge variable was computed as a 2D gradient of the percentage of canopy cover, over a 3×3 neighbourhood [11]:

$$\frac{\mathrm{d}Cover}{\mathrm{d}x} = ((c + 2f + i) - (a + 2d + g)/(8 * 25) \tag{6}$$

$$\frac{\mathrm{d}Cover}{\mathrm{d}y} = ((g + 2h + i) - (a + 2b + c))/(8 * 25) \tag{7}$$

$$Edge = \sqrt{(\frac{dz}{dx})2 + (\frac{dz}{dy})2]} \tag{8}$$

where x and y are the latitudinal and longitudinal directions, and $\{a, b, c, d, e, f, g, h, i\}$ are the values of the percentage of canopy cover in the cell of coordinates $\{(1,1); (1,2); (1,3); (2,1); (2,2); (2,3); (3,1); (3,2); (3,3)\}$ when considering a Moore neighbourhood) around the cell of interest. 25 is the dimension of a cell (in meters).

References

[1] Johnson C, Parker K, Heard D (2001) Foraging across a variable landscape: behavioral decisions made by woodland caribou at multiple spatial scales. Oecologia 127: 590–602.

[2] Weclaw P (2001) Modeling the future of woodland caribou in northern Alberta. Ph.D. thesis, University of Alberta, Canada.

[3] Holleman D, Luick J, White R (1979) Lichen intake estimates for reindeer and caribou during winter. The Journal of Wildlife Management 43: 192–201.

[4] Courtois R (2003) La conservation du caribou forestier dans un contexte de perte d'habitat et de fragmentation du milieu. Ph.D. thesis, Université du Québec à Rimouski, Canada.

[5] Boertje R (1985) An energy model for adult female caribou of the Denali herd, Alaska. Journal of Range Management 38: 468–473.

[6] Boertje R (1990) Diet quality and intake requirements of adult female caribou of the Denali Herd, Alaska. Journal of Applied Ecology 27: 420–434.

[7] Stelfox J, Adamczewski J (1993) Hoofed mammals of Alberta. Lone Pine Pub.

[8] Van Moorter B, Visscher D, Benhamou S, Börger L, Boyce M, et al. (2009) Memory keeps you at home: a mechanistic model for home range emergence. Oikos 118: 641–652.

[9] Howery L, Bailey D, Laca E (1999) Impact of spatial memory on habitat use. Grazing behavior of livestock and wildife 70: 91–100.

[10] Hebblewhite M, Merrill E (2009) Trade-offs between predation risk and forage differ between migrant strategies in a migratory ungulate. Ecology 90: 3445–3454.

[11] Burrough P, McDonnell R, Burrough P, McDonnell R (1998) Principles of geographical information systems, volume 333. Oxford university press, Oxford.
